# Metatranscriptomics Reveals Antibiotic-Induced Resistance Gene Expression in the Murine Gut Microbiota

**DOI:** 10.3389/fmicb.2020.00322

**Published:** 2020-03-06

**Authors:** Benjamin J. Korry, Damien J. Cabral, Peter Belenky

**Affiliations:** Department of Molecular Microbiology and Immunology, Division of Biology and Medicine, Brown University, Providence, RI, United States

**Keywords:** antibiotic resistance genes, microbiome, resistome, antibiotics, metagenomics, metatranscriptomics

## Abstract

Antibiotic resistance is a current and expanding threat to the practice of modern medicine. Antibiotic therapy has been shown to perturb the composition of the host microbiome with significant health consequences. In addition, the gut microbiome is known to be a reservoir of antibiotic resistance genes. Work has demonstrated that antibiotics can alter the collection of antibiotic resistance genes within the microbiome through selection and horizontal gene transfer. While antibiotics also have the potential to impact the expression of resistance genes, metagenomic-based pipelines currently lack the ability to detect these shifts. Here, we utilized a dual sequencing approach combining shotgun metagenomics and metatranscriptomics to profile how three antibiotics, amoxicillin, doxycycline, and ciprofloxacin, impact the murine gut resistome at the DNA and RNA level. We found that each antibiotic induced broad, but untargeted impacts on the gene content of the resistome. In contrast, changes in ARG transcript abundance were more targeted to the antibiotic treatment. Doxycycline and amoxicillin induced the expression of tetracycline and beta-lactamase resistance genes, respectively. Furthermore, the increased beta-lactamase resistance gene transcripts could contribute to an observed bloom of *Bacteroides thetaiotaomicron* during amoxicillin treatment. Based on these findings, we propose that the utilization of a dual sequencing methodology provides a unique capacity to fully understand the response of the resistome to antibiotic perturbation. In particular, the analysis of transcripts reveals that the expression and utilization of resistance genes is far narrower than their abundance at the genomic level would suggest.

## Introduction

Antibiotic resistance has emerged as a major threat to human health. In the United States, millions of people suffer from infections caused by antibiotic resistant bacteria, and tens of thousands die as a result (CDC, 2013). Although antibiotic resistance is recognized to be an ancient phenomenon predating the therapeutic use of antibiotics (D’Costa et al., 2011; Bhullar et al., 2012; Santiago-Rodriguez et al., 2015), recent misuse and overuse of antibiotics has led to an increase in the selection for antibiotic resistance genes (ARGs) and has contributed to the spread of infections caused by antibiotic resistant bacteria. Thus, it is important to understand how antibiotic exposure impacts the abundance and expression of resistance genes in the host. Culture-independent methods of profiling entire microbial communities for ARGs have greatly expanded our ability to detect and track resistance elements utilizing high-throughput sequencing techniques (Sommer et al., 2009; Lu et al., 2014; Crofts et al., 2017; Arango-Argoty et al., 2018; Yin et al., 2018). This important development in detection has led to the discovery of ARGs in gut colonizing microbes.

The gut microbiota is now recognized as an important element in human health and disease (Cho and Blaser, 2012; Human Microbiome Project Consortium, 2012; Pflughoeft and Versalovic, 2012; Lynch and Pedersen, 2016), and can serve as a reservoir of antibiotic resistant bacteria (Sommer et al., 2010). Research into the collection of ARGs within the microbiome, termed the “resistome”(D’Costa et al., 2006; Wright, 2007), has begun to explore resistance genes within the gut. Studies have characterized the gut resistome in terms of composition (Sommer et al., 2009, 2010; Hu et al., 2013), life history (Lu et al., 2014; Moore et al., 2015; Parnanen et al., 2018), geographic location (Forslund et al., 2013; Hu et al., 2013; Pehrsson et al., 2016), antibiotic perturbation (Jernberg et al., 2007; Looft et al., 2012), and other factors. In addition, horizontal gene transfer (HGT) of ARGs between bacteria in the gut has been theorized to contribute to the spread of resistance (Shoemaker et al., 2001; Lester et al., 2006; Karami et al., 2007; Smillie et al., 2011; Stecher et al., 2012; Huddleston, 2014). Since the gut microbiome is a reservoir of antibiotic resistance and has the potential to promote HGT of ARGs, it is of particular importance to understand the role of antibiotics in shaping the landscape of antibiotic resistance in this microbial environment.

Antibiotic therapy has been shown to have a dramatic impact on the microbiome, playing a role in gut dysbiosis (Dethlefsen et al., 2008; Dethlefsen and Relman, 2011; Becattini et al., 2016), increasing susceptibility to infection (Ubeda et al., 2010; Buffie et al., 2012), and altering the composition of the gut resistome (Jernberg et al., 2007; Zhang et al., 2013; Palleja et al., 2018). Less is known about how antibiotics promote ARG selection *in vivo* (Sjolund et al., 2003; Looft et al., 2012), and the impact of antibiotics on the expression of resistance genes in the host microbiota. In response to antibiotic treatment, changes in the resistome may be stochastic, induced by the underlying changes in population structure, or more directed and targeted toward the drug administered. Here we utilize a dual sequencing methodology that employs both metagenomics and metatranscriptomics to reveal broad, untargeted changes in the resistome at the DNA level and a narrower, drug-specific response at the RNA level.

## Materials and Methods

### Mouse Experiments

In a previous study, we obtained total cecal DNA and RNA from six-week-old female C57BL/6J mice that were treated with amoxicillin (0.1667 mg/mL) for 12 h, or ciprofloxacin (0.0833 mg/mL), or doxycycline hydrochloride (0.067 mg/mL) for 24 h (Cabral et al., 2019). All antibiotic treatments were administered in drinking water, which was provided *ad libitum*. Based on the estimate that a healthy mouse drinks 150 mL/kg of water per day, these concentrations were selected to administer a dosage of 25 (amoxicillin), 12.5 (ciprofloxacin), or 10 (doxycycline) mg/kg/day (Yang et al., 2017). Control mice were provided with pH-matched water. Each group had four mice that were split into at least two cages per group to account for cage effects. After the 12- or 24-h treatments, mice were sacrificed and cecal contents were collected and stored in DNA/RNA Shield Collection and Lysis Tube from Zymo Research (Irvine, CA, United States). Samples were kept on ice prior to being transferred to −80°C for permanent storage. Mouse experiments were carried out at the Brown University mouse facility with approval from the Institutional Animal Care and Use Committee of Brown University.

### Nucleic Acid Extraction and Quantification

DNA and RNA were extracted from cecal samples using the ZymoBIOMICS DNA/RNA Miniprep Kit from Zymo Research (Irvine, CA, United States), eluted in nuclease-free water, and stored at −80°C. Extracted DNA and RNA were quantified using the dsDNA-BR and RNA-HS kits on a Qubit^TM^ 3.0 Fluorometer (Thermo Fisher Scientific, Waltham, MA, United States).

### Metagenomic and Metatranscriptomic Library Preparation

Metagenomic libraries were prepared using the Ovation^®^ Ultralow System V2 kit from NuGEN (San Carlos, CA, United States). DNA was sheared to a median fragment size of 300 bp using a Covaris S220 High Performance Ultrasonicator (Woburn, MA, United States), and used to prepare metagenomic libraries following the manufacturer’s protocol. Metatranscriptomic libraries were prepared using Ovation^®^ Complete Prokaryotic RNA-seq Library System from NuGEN. Extracted RNA was first treated with DNA rDNase I to remove contaminating DNA. Next, host mRNA and bacterial ribosomal RNA was reduced using the MICROBEnrich and MICROBExpress kits from Invitrogen (Carlsbad, CA, United States). This processed RNA was then used to generate metatranscriptomic libraries following the manufacturer’s protocol with the addition of AnyDeplete probes designed to specifically remove fragments originating from murine osteosarcoma virus, a known source of contamination sequences.

### Sequencing

Metagenomic and metatranscriptomic libraries were sequenced on an Illumina HiSeqX using paired-end, 150 bp reads. Sequencing yielded an average of 26,113,145 (± 11,436,616) and 85,599,941 (± 11,674,614) raw reads per metagenomic and metatranscriptomic libraries, respectively. All reads were deposited in the NCBI Short Read Archive under BioProject numbers PRJNA504846 (metagenomics) and PRJNA515074 (metatranscriptomics). This data set was previously published by Cabral et al. (2019). The DNA, RNA, and subsequent libraries and sequencing data were the same as those initially published in Cabral et al. (2019). Here, we reanalyze this data using a different set of pipelines and with a focus on the detection of ARGs.

### Processing of Raw Reads

Raw reads from both metagenomic and metatranscriptomic sequencing were processed using the kneadData wrapper script (McIver et al., 2018). Reads were trimmed with Trimmomatic (version 0.36) with SLIDINGWINDOW set at 4:20, MINLEN set at 50, and ILLUMINACLIP: TruSeq3-PE.fa:2:20:10 (Bolger et al., 2014). Sequences from contaminating C57BL/6NJ mouse genome and two murine retroviruses [murine osteosarcoma virus (accession NC_001506.1) and mouse mammary tumor virus (accession NC_001503)] were filtered out using Bowtie2 (Langmead and Salzberg, 2012). In addition to this preprocessing, bacterial ribosomal reads were removed from the datasets using the SILVA 128 database (Pruesse et al., 2007). Based on the principle coordinate analysis (PCoA) of the metatranscriptomic derived resistomes we determined that doxycycline sample 1 was an outlier and it was removed from further analysis (Supplementary Figure S1). Doxycycline sample 1 had roughly 10 times the number of ARG hits relative to all other samples despite it being sequenced to a similar depth (Supplementary Table S1).

### Taxonomic Analysis of Metagenomic Reads

Cleaned metagenomic forward reads were classified against a database containing all prokaryotic genomes downloaded from NCBI RefSeq using Kaiju (version 1.7.0) using the MEM run mode and the default cutoffs for *E*-value and minimum required match length (Menzel et al., 2016) (full relative abundance tables can be found in Supplementary Table S2). The taxonomic output table was analyzed in R (version 3.5.2) using the phyloseq package (version 1.24.2) to calculate alpha and beta diversity metrics (McMurdie and Holmes, 2013). PCoA was performed using the Bray-Curtis Dissimilarity metric (Bray and Curtis, 1957).

### Metagenomic Assembly and Binning

The PATRIC web server (3.5.43) (Wattam et al., 2017) was utilized to bin and assign taxonomy to contigs assembled using metaSPAdes within SPAdes (3.13.0) (Nurk et al., 2017).

### Antibiotic Resistance Gene Analysis

Processed reads were joined using the fastq-join function of the ea-utils package (Aronesty, 2011). The joined reads were then queried for antibiotic resistance genes using DeepARG (version 1) (Arango-Argoty et al., 2018) using the default settings (0.8 minimum coverage of alignment, *E*-value cutoff 1e-10, 50% minimum percentage of identity) and excluding “predicted” resistance genes (full counts tables of ARGs and ARG classes can be found in Supplementary Table S3). ARGs at the DNA level from the metagenomic data were identified by running cleaned, merged PE reads through the DeepARG pipeline. On average 45,768 (± 10,213) ARG hits were obtained from an average of 26,281,760 (± 5,410,439) cleaned paired-end (merged forward and reverse read files). Using the same method to identify ARGs at the RNA level from metatranscriptomic sequencing, we found an average of 14,576 (± 6,147) ARG hits from an average of 49,338,997 (± 10,392,548) cleaned paired-end (merged forward and reverse read files) reads.

### Statistical Analyses and Figure Generation

Differential abundance of ARGs and ARG classes between treatments and controls was determined using DESeq2 (version 1.20.0) (Love et al., 2014) in R (version 3.5.2) using default parameters. Statistical differences between alpha diversities were calculated in GraphPad Prism (version 8.0) (GraphPad Software, La Jolla, CA, United States). All figures were generated using Prism 8.0, except Supplementary Figure S4 which was generated using the Clustal Omega tool (Sievers and Higgins, 2018) on the EMBL-EBI web server (Madeira et al., 2019).

## Results and Discussion

### Microbial Diversity

**FIGURE 1:**
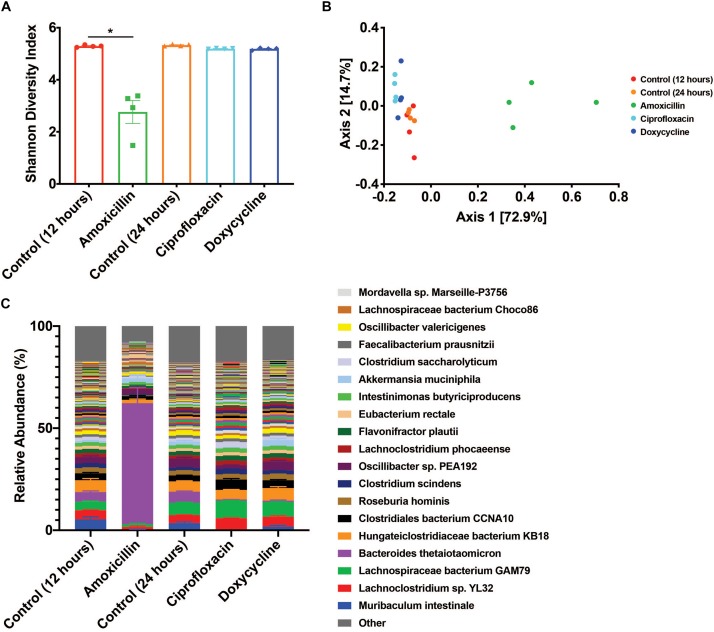
Antibiotic treatment has variable impacts on the diversity and taxonomic structure of the microbiome. **(A)** Shannon diversity index displayed as mean ± SEM (*p < 0.05 Mann-Whitney U test, *n* = 4). **(B)** PCoA based on Bray-Curtis beta diversity. **(C)** Relative abundance of bacterial species displayed as mean ± SEM (*n* = 4, top 250 most abundant species colored, full relative abundance table available in Supplementary Table S2).

Antibiotic treatment was administered for either 12 h for amoxicillin (beta-lactam) or 24 h for ciprofloxacin (fluoroquinolone) and doxycycline (tetracycline) with untreated time-matched controls. The microbiome of the amoxicillin treated mice displayed a marked reduction in bacterial alpha diversity (*p* < 0.05, Mann-Whitney U test), however, no change in diversity was observed in mice treated with ciprofloxacin or doxycycline (Figure 1A). Shifts in beta diversity were observed between each treatment and their respective controls indicating that these antibiotics elicit unique changes to the murine gut microbiome at a taxonomic level (*p* < 0.05, PERMANOVA) (Figure 1B). Perhaps the most drastic taxonomic change was the expansion of *Bacteroides thetaiotaomicron* in the microbiota of amoxicillin treated mice (Figure 1C and Supplementary Figure S2). Overall, we found that out of the three antibiotics tested, amoxicillin had the most profound impact on the murine cecal microbiome community in terms of diversity and species relative abundance, while ciprofloxacin and doxycycline exhibit less drastic changes. A more detailed description of the taxonomic shifts is detailed in Cabral et al. (2019), while in this study we focus on the ARGs.

### ARG Abundance

While metagenomic analysis is commonly used to characterize the resistome, it can only report the genes found in the community but does not provide information about the actual expression of those genes. In the metagenomic data, we determined the average number of ARGs relative to total reads to calculate the relative abundance of ARGs in the community. There were 1.74E-03 (± 1.62E-04) ARGs per read detected in the metagenomic data (Supplementary Table S1), with an average of 336 (± 14) unique ARGs found in each metagenomic sample (Supplementary Figure S3A). In contrast to the metagenomic data, metatranscriptomics cannot describe the structure of the community, but it can identify the portion of the total genes actively transcribed by the microbiome. In the metatranscriptomic data, there were 3.1E-04 (± 1.67E-04) ARGs per read (Supplementary Table S1), with an average of 161 (± 40) unique ARGs found in each metatranscriptomic sample (Supplementary Figure S3B). This data shows that there are more unique ARGs found in the DNA than in the RNA despite the higher sequencing depth used for metatranscriptomics. This discrepancy could indicate that many of the ARGs encoded in the microbiome are not actively transcribed with or without drug pressure.

### Resistome Diversity

**FIGURE 2:**
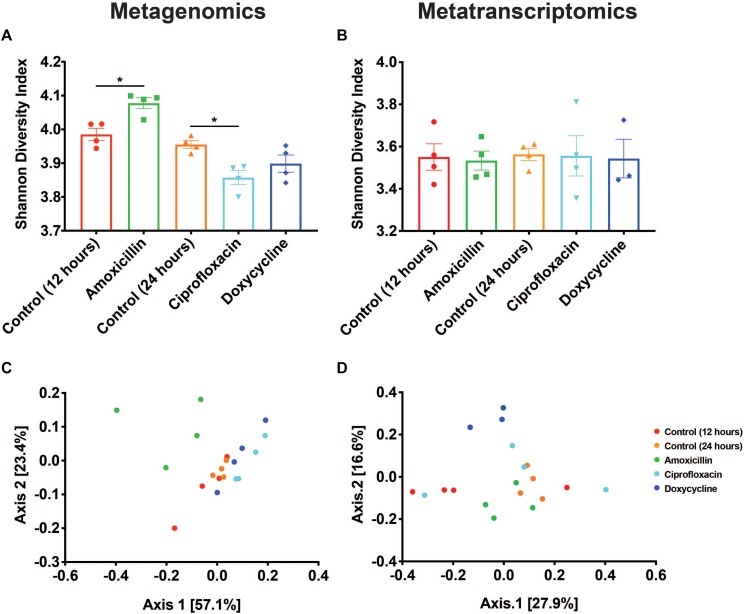
Antibiotics have variable impacts on the diversity and structure of the resistome. **(A,B)** Shannon diversity index based on resistance gene counts displayed as mean ± SEM for both metagenomic and metatranscriptomic data (*p < 0.05 Mann-Whitney U test). **(C,D)** PCoA based on Bray-Curtis of resistance gene counts for both metagenomic and metatranscriptomic data.

Two of the antibiotics examined, amoxicillin and ciprofloxacin, had unique impacts on the taxonomic composition of the microbiome resulting in corresponding shifts in the resistome diversity at the DNA level. Metagenomic data showed that compared to time-matched controls, there was a significant increase in the Shannon diversity of the resistomes with amoxicillin (*p* < 0.05, Mann-Whitney U test), and a decrease in the Shannon diversity with ciprofloxacin (*p* < 0.05, Mann-Whitney U test), while doxycycline treatment had no impact (Figure 2A). Analysis of the Bray-Curtis beta diversity revealed significant differences in amoxicillin and ciprofloxacin groups compared to their time-matched controls (*p* < 0.05, PERMANOVA), but not in the doxycycline treated group (*p* = 0.2, PERMANOVA) (Figure 2C). However, these shifts in ARG diversity profiles did not necessarily reflect a drug specific selection but rather resulted from the overall shift in microbiome composition. We found that while treatment induced changes in alpha diversity of the resistome at the DNA level, it did not impact resistome alpha diversity at the RNA level (Figure 2B). The lower and more stable alpha diversity of the RNA reads compared to the DNA reads likely stems from the fact that many of the genes detected in the metagenomics are not actively transcribed under vehicle or antibiotic treatment. We also found that amoxicillin and ciprofloxacin did not significantly impact Bray-Curtis ARG diversity at the RNA level (*p* = 0.128, *p* = 0.397, PERMANOVA). Additionally, in the metatranscriptomic data, doxycycline did induce a significant Bray-Curtis shift from the 24-h controls (*p* < 0.05, PERMANOVA) (Figure 2D). This shift in beta-diversity may be driven by the induction of drug targeted ARG transcripts observed in the doxycycline treated samples.

### ARG Class Level Changes in Response to Antibiotics

**FIGURE 3:**
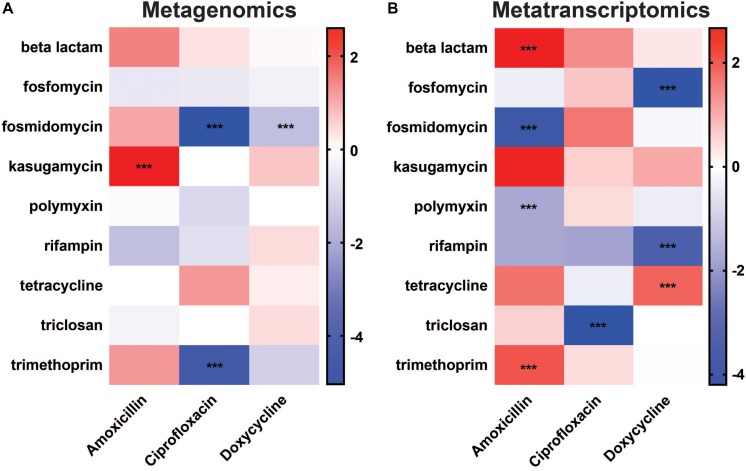
Differential abundance of antibiotic resistance gene classes. Changes in ARG class abundances after antibiotic treatment observed in **(A)** metagenomic and **(B)** metatranscriptomic data. The color scale represents log_2_ fold change (***log_2_FC ≥ 1.5/ ≤ –1.5 and *padj* < 0.05) (Full ARG class counts available in Supplementary Table S3).

Antibiotic-induced shifts in ARG classes were determined using DESeq2 and considered significant with a log_2_FC ≥ 1.5 or ≤ −1.5 and an adjusted *p*-value < 0.05 (Figure 3A). At the DNA level, we did not find any changes in ARG classes that directly corresponded to antibiotic treatment. For example, the beta-lactam resistance class was not increased with amoxicillin. Instead, we report that the only significant changes in ARG classes were an increase in kasugamycin class ARGs in response to amoxicillin treatment, decreases in the fosmidomycin and trimethoprim classes in response to ciprofloxacin treatment, and a decrease in the fosmidomycin class in response to doxycycline treatment (Figure 3A). As a whole, none of the treatments led to an induction of ARG classes targeted toward the drug administered.

In contrast to the lack of drug targeted changes at the DNA level, metatranscriptomic sequencing showed an induction of ARG classes targeted to two of the treatments at the RNA level (Figure 3B). Overall, there were a number of significant changes in ARG classes against beta-lactams, fosmidomycin, polymyxin, and trimethoprim in response to amoxicillin treatment, triclosan in response to ciprofloxacin treatment, and fosfomycin, rifampin, and tetracycline in response to doxycycline treatment. Most interestingly, amoxicillin significantly increased the abundance of the beta-lactam ARG class with log_2_FC 2.67 (*padj* = 2.50E-04), and doxycycline increased the abundance of the tetracycline ARG class with log_2_FC 1.81 (*padj* 3.10E-21) in the RNA (Figure 3B). Thus, in contrast to the metagenomic data, metatranscriptomics shows a significant increase in ARG classes that are targeted to the antibiotic treatment and have the potential to provide a fitness advantage to members of the gut microbiota.

### ARG Level Changes in Response to Antibiotics

**FIGURE 4:**
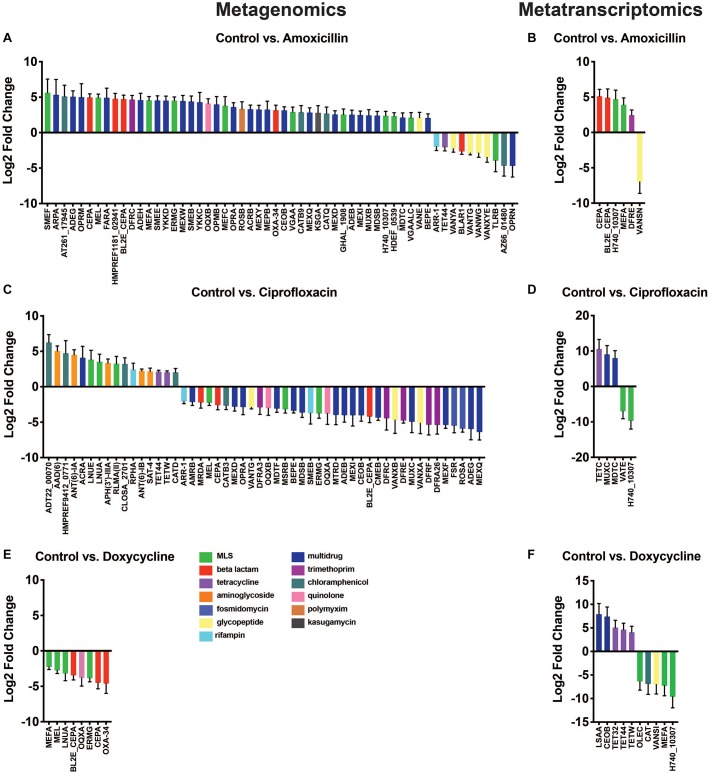
Differential abundance of antibiotic resistance genes. Changes in ARG abundances after antibiotic treatment observed in metagenomic **(A,C,E)** and metatranscriptomic data **(B,D,F)**. Bars represent change in gene/transcript abundance after exposure to antibiotics displayed as log_2_ fold change ± standard error (log_2_FC ≥ 1.5/ ≤ –1.5 and *padj* < 0.05) (Full ARG class counts available in Supplementary Table S3).

Results from the differential abundance analysis show that the antibiotics tested have variable impacts on the abundance of AR genes and transcripts. At the metagenomic level, we found a set of differentially abundant genes that appeared general and unrelated to the antibiotic utilized. In contrast, the transcriptional response was much narrower and in the case of amoxicillin and doxycycline, it appears that antibiotic therapy promoted genes directly targeted to the drug utilized. This dichotomy between DNA and RNA level responses could not have been detected without using a dual sequencing approach. Overall, there were fewer differentially abundant ARG transcripts (21 transcripts) found in the metatranscriptomic analysis compared to the number of differentially abundant ARGs (116 genes) in the metagenomic data (Figures 4A–F). This is a reflection of the fewer ARG reads found in the metatranscriptomic data, as well as the more specific response of the microbiome at a transcriptional level compared to the broad metagenomic changes. This is best exemplified by changes in ARGs targeted to antibiotics, specifically amoxicillin and doxycycline.

While the observations made at the ARG class level were fairly broad, the gene level data provided more insights into the direct impact of antibiotic administration on specific ARGs. The differential expression tool DESeq2 was used to analyze the antibiotic-induced changes in AR gene and transcript abundance (Figure 4A). We found 56 significantly elevated or reduced ARGs after amoxicillin treatment. Of these 56, two beta-lactamase genes of interest, *cepA* and *bl2e_cepA*, had significant increases in gene abundances of log_2_FC 4.96 (*padj* = 4.10E-20) and log_2_FC 4.73 (*padj* = 6.72E-16), respectively. In addition to drug targeted genes, we also found increases in a much larger set of untargeted genes (42 genes). It is possible that these changes are the result of taxonomic shifts in bacteria that encode these genes, rather than a direct selection promoted by the induced resistance genes. The beta-lactamase genes increased in the metagenomic data are also increased at the RNA level, highlighted by significantly higher transcript abundances of log_2_FC 5.12 (*padj* = 2.40E-05), 4.93, (*padj* = 2.01E-3), for *cepA* and *bl2e_cepA*, respectively (Figure 4B). This may suggest that in response to amoxicillin the community increased transcription of beta-lactamase genes leading to increased bacterial fitness. Conversely, it is also possible that this change merely reflects a bloom in the bacterium encoding these transcripts rather than a direct transcriptional response.

To identify the bacterial origin of the beta-lactamase genes found in our dataset, bacterial genomes were assembled from metagenomic data. Within the *B. thetaiotaomicron* metagenomically assembled genome (MAG), we identified a region corresponding to a class A beta-lactamase gene with 100% protein sequence homology to a subclass A2 beta-lactamase. Due to its high degree of sequence similarity, this gene likely corresponds to the reads assigned to the *cepA* and *bl2e_cepA* genes (Supplementary Figure S4). The relative bloom *of B. thetaiotaomicron* after amoxicillin treatment may account for the increase in both the *cepA* and *bl2e_cepA* gene abundance and transcript level abundance. It is possible that the survival of this taxa during amoxicillin treatment may be promoted by these genes, although we cannot make a definitive conclusion without more evidence. Various laboratory strains and patient isolates of *Bacteroidales* have been shown to exhibit high levels of resistance to beta-lactams including amoxicillin (Rogers et al., 1994; Nakano et al., 2011; Stentz et al., 2015). Previous research into *B. thetaiotaomicron* found that this bacterium produces outer membrane vesicles (OMVs) containing cephalosporinase enzymes that protect neighboring bacteria from beta-lactam antibiotics (Stentz et al., 2015). Because *B. thetaiotaomicron* is a common human commensal (Curtis et al., 2014; Banerjee et al., 2018), this work has interesting implications for the complex microbial environment of the gut microbiome where *B. thetaiotaomicron* could have a role in modulating antibiotic activity across many taxa through OMV-secreted beta-lactamase enzymes.

Metagenomic data showed that ciprofloxacin treatment induced significant changes in ARG abundance, most notably an increase in the relative abundance of several chloramphenicol, aminoglycoside, and MLS class genes, log_2_FC > 1.5 (*padj* < 0.05), and a decrease in the abundance of genes related to multidrug and fosmidomycin resistance log_2_FC < −1.5 (*padj* < 0.05) (Figure 4C). No significant increases in fluoroquinolone resistance genes were found in this dataset, however, it should be noted that point mutations conferring resistances were not included in the ARG database used. Thus, fluoroquinolone resistance mutations in *gyrA*, *gyrB*, or *parC* could not be identified from this analysis and might be present in the resistome. No ARG transcripts corresponding to fluoroquinolone resistance were significantly elevated in the ciprofloxacin treated samples. The transcripts of *tetC*, *muxC*, and *mdtC*, all genes encoding efflux system components, were significantly increased by ciprofloxacin treatment (log_2_FC ≥ 1.5, *padj* < 0.05) (Figure 4D). Although none of these genes have been shown to directly efflux ciprofloxacin, the fact that fluoroquinolone treatment exclusively increased transcription of efflux type ARGs remains interesting.

At the DNA level, no genes were increased in abundance during doxycycline treatment; however, several were decreased in abundance. Although genes known to confer doxycycline resistance were detected in the metagenomic data (Supplementary Table S3), they were not significantly changed due to doxycycline treatment (Figure 4E). While doxycycline did not increase the abundance of any tetracycline class ARGs in the metagenomic data set, we did detect changes in the metatranscriptomic data. At the RNA level, doxycycline appears to have a targeted effect on the transcript abundance of tetracycline resistance genes with significant increases in the abundance of *tet*32, *tet*44, and *tetW* with log_2_FCs of 5.06, 4.63, and 4.07, respectively (*padj* = 1.6E-2, 1.2E-2, 2.2E-2, respectively) (Figure 4E). This distinct difference in gene versus transcript abundance of these tetracycline class ARGs suggests that while short-term treatment with doxycycline may not select for bacteria encoding these resistance genes, it may induce their expression. All three tetracycline resistance genes with increased transcript abundance, *tet*32, *tet*44, and *tetW* have been shown to offer protection to tetracycline antibiotics through ribosomal protection mechanisms (Melville et al., 2001; Stanton et al., 2004; Abril et al., 2010). The increased transcript abundance of several *tet* genes in response to doxycycline, combined with unchanged levels of these same *tet* genes in the metagenomic dataset, suggests that their elevated transcriptional activity may be providing protection and enabling the population of *tet*-carrying bacteria to remain stable during treatment. The doxycycline-induced expression of several tetracycline resistance genes highlights the need for increased transcriptional profiling of ARGs. Relying solely on metagenomics without utilizing metatranscriptomic sequencing, we would have missed this ARG activity that may contribute to bacterial survival during antibiotic pressure.

## Conclusion

In this study, we show that short-term antibiotic pressure leads to the differential expression of specific ARGs within the microbiome, but alters the metagenomic landscape in a less targeted way. We use both shotgun metagenomics and metatranscriptomics to profile how three antibiotics, amoxicillin (beta-lactam), doxycycline (tetracycline), and ciprofloxacin (fluoroquinolone), impact the diversity, composition, and transcriptional response of the murine gut resistome. We found that combining these two sequencing methods provides unique perspectives on ARGs in the microbiome that would have been missed by using metagenomics exclusively. For example, we found that at the RNA level a majority of the induced ARGs were targeted against the administered antibiotic, while at the DNA level we found more changes overall, but we did not find a drug-specific pattern. Our results show that both bactericidal and bacteriostatic antibiotic treatment alters the resistome at both the RNA and DNA level, but that these changes may be more specific to the drug administered at the transcript abundance level.

This work highlights the impacts of three antibiotics on the murine cecal resistome as well as the importance of both metagenomic and metatranscriptomic profiling of ARGs. However, due to the current methodology, there are several limitations to this work that must be considered. Experiments were done in mice in a closed mouse facility with reduced exposure to environmental bacteria and the antibiotic exposure period was fairly short. Both of these factors will reduce the opportunity for new ARGs to enter the microbiome and for selection to act on existing ARGs. In addition, due to limitations in strain level identification from current metagenomic methodologies, we are unable to predict whether or not there was selection of specific ARG containing strains following antibiotic treatment. Additionally, the computational pipeline utilized to detect ARGs is unable to identify point mutations and their contributions to the resistome. These aspects limit our ability to detect selective events and the evolution of resistance. Finally, these experiments were conducted with a limited sample size (*n* = 4), and no samples were collected at time 0. These two factors may limit our ability to detect smaller shifts in ARG levels with sufficient significance and may hinder the detection of baseline changes to the microbiota of the control mice over the course of the experiment.

Despite these limitations, we believe that the dual sequencing approach has real benefits over purely DNA-based approaches. The widespread use of common antibiotics such as those tested in this study contributes to the dissemination of resistance genes in the human population and our microbiomes. However, as demonstrated here, the presence of an ARG in the metagenome may not necessarily indicate that it will be transcribed at baseline or in response to antibiotics. Thus, as we continue to develop strategies to monitor resistance in patient populations it will be important to track both gene presence and expression.

## Data Availability Statement

Raw metagenomic and metatranscriptomic reads were deposited in the NCBI Short Read Archive under the BioProject number PRJNA504846.

## Ethics Statement

The animal study was reviewed and approved by Institutional Animal Care and Use Committee of Brown University.

## Author Contributions

BK, DC, PB contributed to the conception and design of the study. DC performed the experiments and sample processing. BK performed the data analysis and drafted the manuscript. BK, DC, and PB contributed to editing drafts of the manuscript.

## Conflict of Interest

The authors declare that the research was conducted in the absence of any commercial or financial relationships that could be construed as a potential conflict of interest.
