# Supplementary material for: Metatranscriptomics Reveals Antibiotic-Induced Resistance Gene Expression in the Murine Gut Microbiota
Source: Front Microbiol. 2020 Mar 6;11:322. doi: 10.3389/fmicb.2020.00322 (PMC7069102; doi:10.3389/fmicb.2020.00322)
Supplement: Supplementary file 1 [file Data_Sheet_1.docx]

**Supplementary Data**

**Table S1 –** Number of cleaned merged reads and ARG hits detected per sample in the metagenomic and metatranscriptomic data. The numbers in parenthesis next to the “control” in the treatment column represent the timepoint at which the control sample was collected (in hours). The numbers after the treatment name (1 - 4) represent the numbering of the four samples in each treatment group.

| **Treatment** | **Clean Reads Metagenomic** | **ARG Hits Metagenomic** | **Clean Reads Metatranscriptomic** | **ARG Hits Metatranscriptomic** |
| --- | --- | --- | --- | --- |
| Control (12) 1 | 22,551,814 | 38,184 | 54,135,535 | 5,368 |
| Control (12) 2 | 14,987,819 | 25,446 | 55,616,863 | 7,874 |
| Control (12) 3 | 27,926,253 | 47,348 | 67,241,907 | 8,087 |
| Control (12) 4 | 23,628,173 | 41,221 | 34,474,417 | 27,594 |
| Amoxicillin 1 | 27,113,593 | 36,827 | 42,340,472 | 14,427 |
| Amoxicillin 2 | 28,594,334 | 47,139 | 41,967,886 | 14,324 |
| Amoxicillin 3 | 36,967,463 | 57,183 | 48,806,177 | 11,350 |
| Amoxicillin 4 | 21,918,209 | 34,402 | 59,681,015 | 16,113 |
| Control (24) 1 | 24,380,522 | 42,049 | 76,421,879 | 18,847 |
| Control (24) 2 | 25,042,390 | 40,697 | 41,795,407 | 14,231 |
| Control (24) 3 | 28,390,347 | 46,533 | 40,949,940 | 16,494 |
| Control (24) 4 | 26,005,446 | 44,284 | 43,928,662 | 16,298 |
| Ciprofloxacin 1 | 21,915,351 | 43,161 | 42,954,907 | 6,257 |
| Ciprofloxacin 2 | 27,536,934 | 54,808 | 46,399,664 | 15,004 |
| Ciprofloxacin 3 | 30,780,514 | 61,164 | 59,725,060 | 13,521 |
| Ciprofloxacin 4 | 23,470,889 | 44,813 | 48,628,764 | 29,332 |
| Doxycycline 1 | 26,777,060 | 48,068 | 40,546,403 | 108,037 |
| Doxycycline 2 | 20,214,974 | 37,301 | 44,226,004 | 14,729 |
| Doxycycline 3 | 39,177,796 | 71,030 | 44,420,451 | 11,237 |
| Doxycycline 4 | 28,255,309 | 53,699 | 43,725,928 | 15,858 |

**
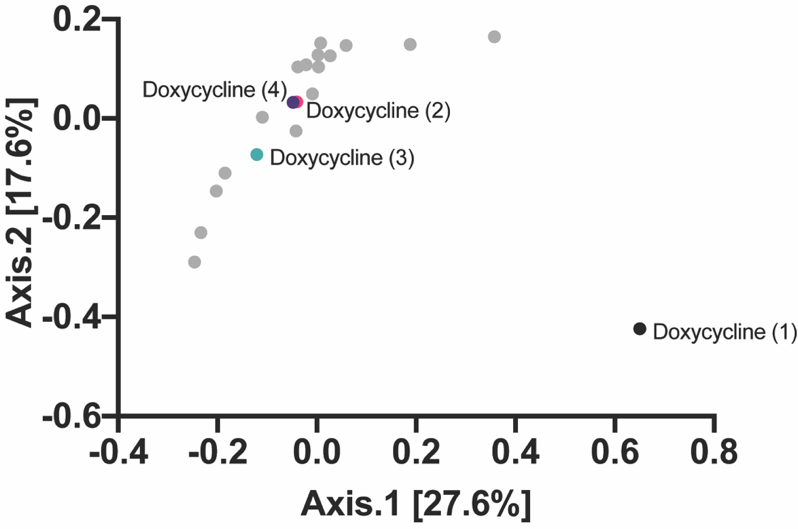
**

**Figure S1 -** PCoA based on Bray-Curtis of resistance gene counts for metatranscriptomic data with the doxycycline samples highlighted.


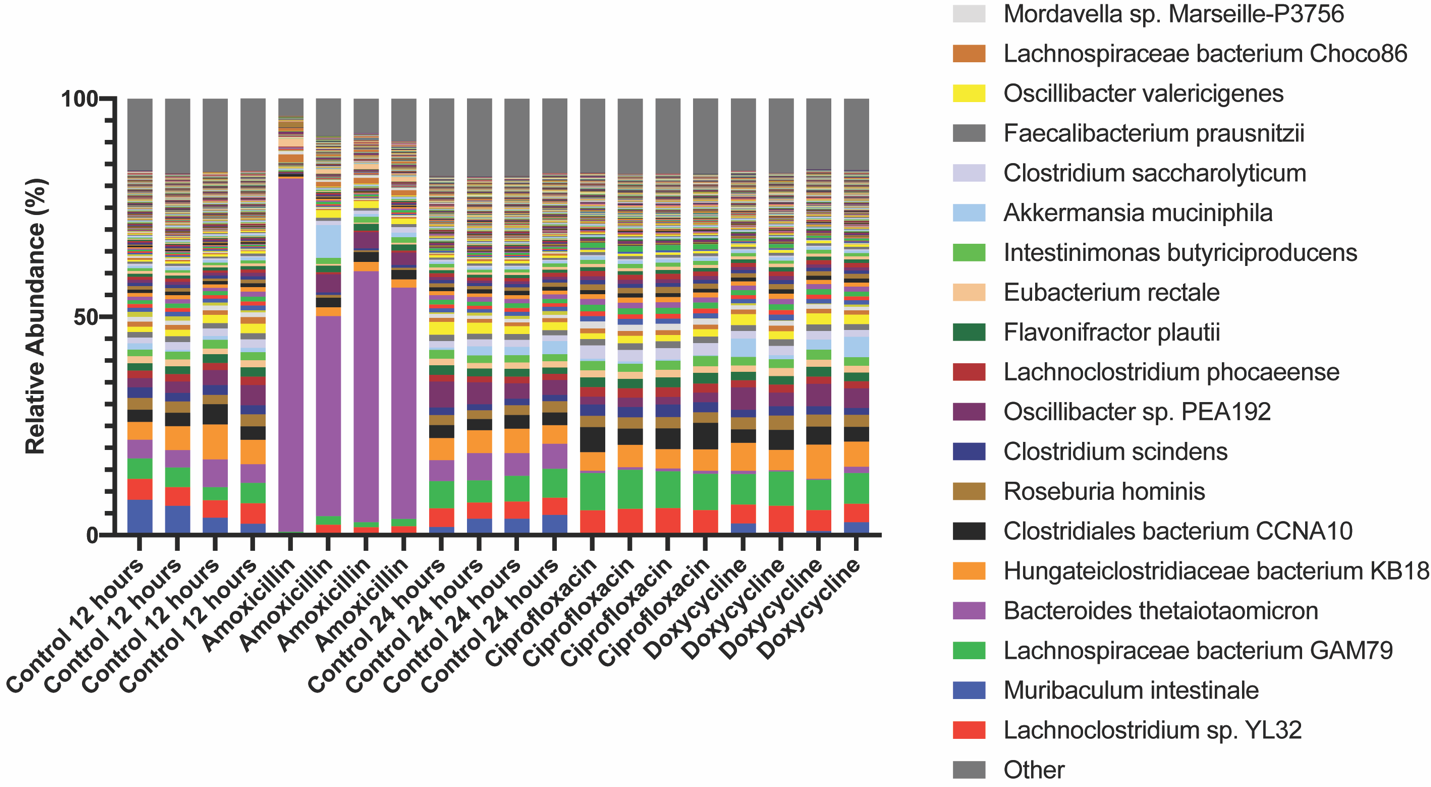


**Figure S2 -** Relative abundance of bacterial species displayed for each metagenomic sample (top 250 most abundant species colored, full relative abundance table available in Table S2).

**
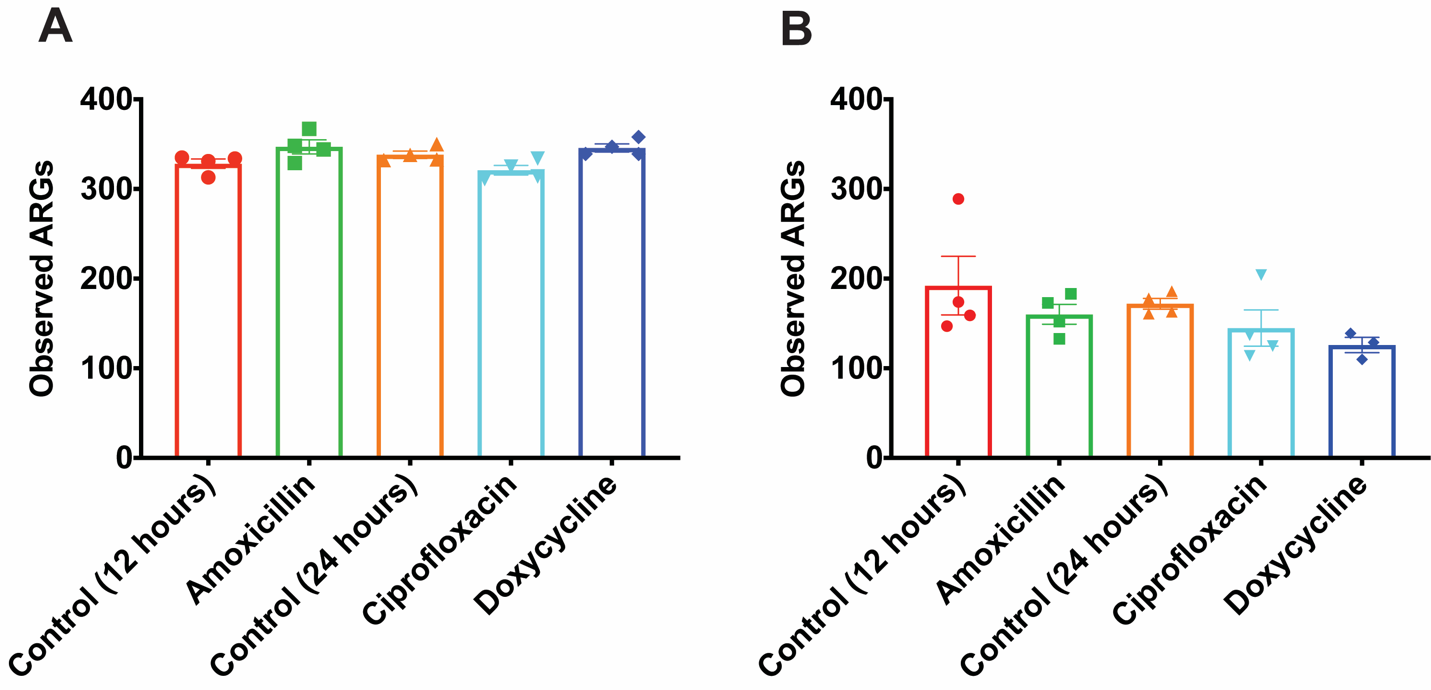
**

**Figure S3 -** Number of unique ARG types observed in both (**A**) metagenomic and (**B**) metatranscriptomic datasets (*p* < 0.05 Mann-Whitney U test).


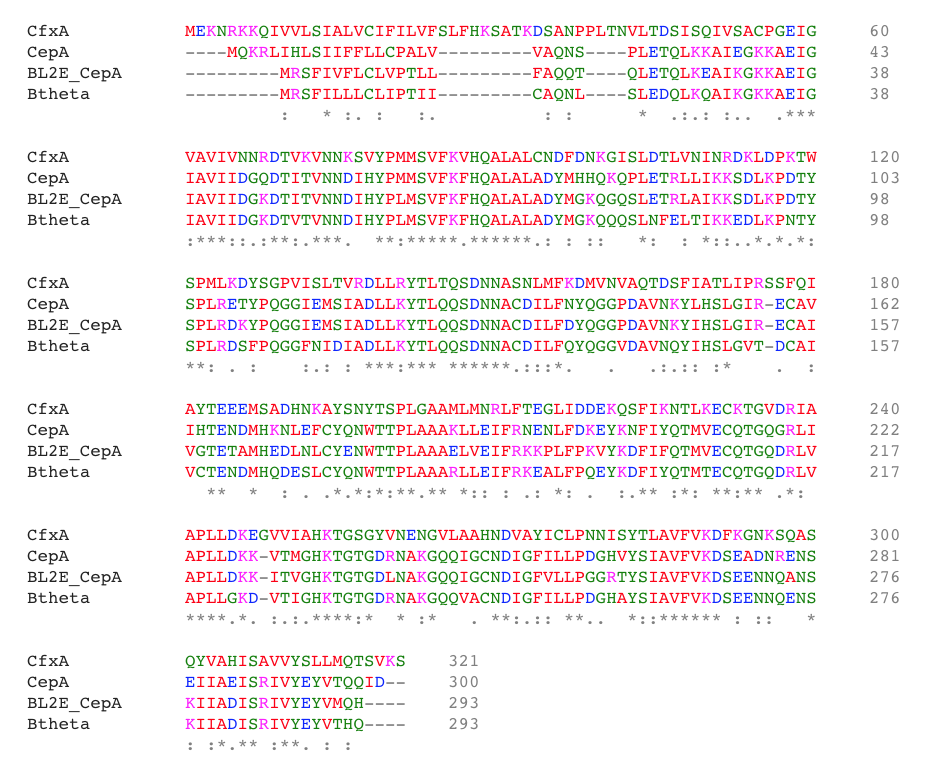


**Figure S4 -** Multiple sequence alignment of *cftxA*, *cepA*, and *bl2e_cepA* protein sequences from the DeepARG V1 database, as well as the subclass A2 beta-lactamase found in the *B. thetaiotaomicron* MAG in this study (labeled Btheta). The alignment was generated using Clustal Omega (1.2.4).

**Table S2** – Species relative abundance for each sample. Tab 1 contains abundances for each bacterial taxa, tab 2 contains the relative abundance of the top 250 most abundant taxa, and tab 3 contains the relative abundance of the top 25 most abundant taxa.

**Table S3 –** Count tables of ARGs and ARG classes for each sample. Tab 1 and 2 delineate ARG and ARG class counts from the metagenomic dataset. Tab 3 and 4 delineate ARG and ARG class counts from the metatranscriptomics dataset.
